# The distribution of covert microbial natural enemies of a globally invasive crop pest, fall armyworm, in Africa: Enemy release and spillover events

**DOI:** 10.1111/1365-2656.13760

**Published:** 2022-06-22

**Authors:** Amy J. Withers, Annabel Rice, Jolanda de Boer, Philip Donkersley, Aislinn J. Pearson, Gilson Chipabika, Patrick Karangwa, Bellancile Uzayisenga, Benjamin A. Mensah, Samuel Adjei Mensah, Phillip Obed Yobe Nkunika, Donald Kachigamba, Judith A. Smith, Christopher M. Jones, Kenneth Wilson

**Affiliations:** ^1^ Lancaster Environment Centre Lancaster University Lancaster UK; ^2^ Rothamsted Research Harpenden UK; ^3^ University of Central Lancashire Preston UK; ^4^ Zambia Agriculture Research Institute Chilanga Zambia; ^5^ Rwanda Agriculture and Animal Resources Development Board Rubona Rwanda; ^6^ University of Cape Coast Cape Coast Ghana; ^7^ University of Zambia Lusaka Zambia; ^8^ Bvumbwe Agricultural Research Station Thyolo Malawi; ^9^ Liverpool School of Tropical Medicine Liverpool UK; ^10^ Malawi‐Liverpool‐Wellcome Trust Clinical Research Programme Blantyre Malawi

**Keywords:** enemy release, invasive, *Metarhizium* *rileyi*, natural enemy, nucleopolyhedrovirus, spillover, *Spodoptera frugiperda*, *Wolbachia*

## Abstract

1. Invasive species pose a significant threat to biodiversity and agriculture world‐wide. Natural enemies play an important part in controlling pest populations, yet we understand very little about the presence and prevalence of natural enemies during the early invasion stages.
2. Microbial natural enemies of fall armyworm *Spodoptera frugiperda* are known in its native region, however, they have not yet been identified in Africa where fall armyworm has been an invasive crop pest since 2016. Larval samples were screened from Malawi, Rwanda, Kenya, Zambia, Sudan and Ghana for the presence of four different microbial natural enemies; two nucleopolyhedroviruses, Spodoptera frugiperda NPV (SfMNPV) and Spodoptera exempta NPV (SpexNPV); the fungal pathogen *Metarhizium rileyi*; and the bacterium *Wolbachia*. This study aimed to identify which microbial pathogens are present in invasive fall armyworm, and determine the geographical, meteorological and temporal variables that influence prevalence.
3. Within 3 years of arrival, fall armyworm was exposed to all four microbial natural enemies. SfMNPV probably arrived with fall armyworm from the Americas, but this is the first putative evidence of host spillover from *Spodoptera exempta* (African armyworm) to fall armyworm for the endemic pathogen SpexNPV and for *Wolbachia*. It is also the first confirmed incidence of *M. rileyi* infecting fall armyworm in Africa.
4. Natural enemies were localised, with variation being observed both nationally and temporally. The prevalence of SfMNPV (the most common natural enemy) was predominantly explained by variables associated with the weather; declining with increasing rainfall and increasing with temperature. However, virus prevalence also increased as the growing season progressed.
5. The infection of an invasive species with a natural enemy from its native range and novel pathogens specific to its new range has important consequences for understanding the population ecology of invasive species and insect–pathogen interactions. Additionally, while it is widely known that temporal and geographic factors affect insect populations, this study reveals that these are important in understanding the distribution of microbial natural enemies associated with invasive pests during the early stages of invasion, and provide baseline data for future studies.

## Graphical abstract

The infection of the invasive fall armyworm, with a natural enemy from its native range and novel pathogens from its new range, has important consequences for understanding the population ecology of invasive species and virus–insect interactions. The authors show that temporal and geographic factors are also important in understanding the distribution of microbial natural enemies.

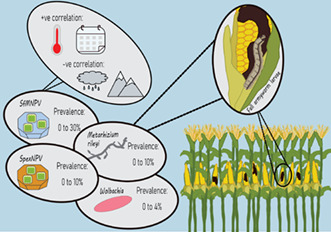

## 1 INTRODUCTION

Invasive species pose a severe threat to both global biodiversity and agricultural sustainability, with countries in sub‐Saharan Africa thought to be among the most vulnerable (Dueñas et al., 2021; Paini et al., 2016). The role of natural enemies in the biocontrol of native crop pests is well documented, where they have been shown to play an important part in regulating the population size of some of the most economically damaging pest species (Cugala et al., 2006). However, the interactions between natural enemies and invasive species are not yet fully understood.

Very little information is known about the way in which natural enemies affect the population dynamics of invasive species, including the potential suppression of invasive pest species in their non‐native range. The expectation is that newly invasive species would conform to the ‘enemy release hypothesis’, which posits that once in a new environment an invasive species is exposed to fewer natural enemies and potential native enemies are not yet familiar with the new host (Cornell & Hawkins, 1993; Torchin & Mitchell, 2004). This can have important implications for the spread of invasive species globally. The loss of microbial natural enemies in the invasive fire ant *Solenopsis invicta*, including the bacterium *Wolbachia*, RNA viruses and fungal pathogens, is likely to have contributed to its rapid spread in three continents (Asia, Australasia and North America) (Yang et al., 2010). However, it has been suggested that the ability to escape from naturally occurring enemies may diminish over time as established invasive species possess up to six times more pathogens compared to more recently introduced invasive species (Mitchell et al., 2010).

Invasive species can be exposed to natural enemies via spillover events, which is when natural enemies of one species target another species that it has recently been exposed to. There is little known about the importance of host spillover events in the presence of natural enemies in invasive species populations because much of the research has focused on the effects of pathogens spilling over from invasive species to native, beneficial insects (Manley et al., 2015; Vilcinskas, 2019). Spillover events are known to cause problems to native insects, such as spillover of the fungal pathogen *Nosema thompsoni* to native ladybirds *Coccinella septempunctata* from the invasive harlequin ladybird *Harmonia axyridus* (Vilcinskas et al., 2013) and spillover of *Nosema ceranae* from invasive honeybees *Apis mellifera* to native stingless bees *Tetragonula hockingsi* (Purkiss & Lach, 2019). Spillover events in insects can be considerable and could contribute to increased natural enemies in invasive species. Evidence from the invasive common wasp *Vespula vulgaris* suggests that its range of microbial natural enemies is similar in its invaded range to its native range because of the spillover of generalist pathogens from native social insects (e.g. other Hymenoptera or *A. mellifera*) (Lester et al., 2015). Therefore, understanding natural enemies, and potential spillover events from native to invasive populations is important as this could have consequences for the population dynamics of invasive species.

There are many other factors that may influence the prevalence and distribution of natural enemies and understanding these can be vital for understanding the invasive hosts' population dynamics. For example, rainfall, humidity and temperature influence the presence of entomopathogenic fungi and nucleopolyhedroviruses (NPVs) (Alyokhin et al., 2011; Fuxa & Richter, 2001; Hajek & Tobin, 2011), which suggests that these pathogens may successfully reduce the population of an invasive species in one location but not another. Interactions between plants, pathogens and herbivorous insect pests mean that the abundance of pathogens in crop pests can be affected by their host plant (Agrawal, 2000; Cory & Hoover, 2006). These interactions occur through many mechanisms, such as insect immune defences changing in response to crop age, plant hormones, prior wounding of the crop and crop type (Ali et al., 1998; Inyang et al., 1998; Shikano et al., 2010; Shikano, McCarthy, et al., 2017; Shikano, Shumaker, et al., 2017).

To address this lack of evidence, this study investigates the extent to which microbial natural enemies can establish themselves in the early phases of a newly invasive host population, and the underlying factors that might increase the risk of exposure for the invasive species. It does this by looking at microbial natural enemies in larval samples of the fall armyworm (*Spodoptera frugiperda*, J.E. Smith, 1797) collected in Africa. Native to the Americas, fall armyworm is an invasive crop pest that spread to Africa in 2016, and by 2018 was present in at least 44 African countries (CABI, 2020; Wilson, 2021). In late 2018 to 2019, it continued its rapid spread into Asia, and then to Australasia in 2020 (CABI, 2020). Fall armyworm is a generalist forager, meaning that it can feed on a wide variety of plants, however, it predominately targets maize in its invaded territories and can cause devastating crop losses (Chhetri & Acharya, 2019; De Groote et al., 2020; Devi, 2018; Pashley, 1986). This leads to economic impacts and can result in severe food shortages, especially in countries where many farmers are smallholders that rely on their crops for subsistence. Improving knowledge of fall armyworm population ecology and control is vitally important in helping to mitigate against this damage, and due to its recent invasion followed by rapid spread, it is an excellent model organism for the study of natural enemies during the early stages of invasions.

In the native range of fall armyworm, there are many different natural enemies present. For example, in Mexico, 30% of larvae collected from maize were targeted by natural enemies, including seven parasitoids, two entomopathogenic fungi and the baculovirus *Spodoptera frugiperda nucleopolyhedrovirus* (SfMNPV) (Virgen et al., 2013). The most common natural enemy of native fall armyworm was SfMNPV, present in 11% of larvae sampled in Nayarit, Mexico (Virgen et al., 2013). Overt SfMNPV disease was present in invasive fall armyworm collected in the Hubei province of China (Lei et al., 2020), and SfMNPV has been isolated from fall armyworm in Nigeria (Wennmann et al., 2021). However, SfMNPV has not yet been reported from elsewhere in Africa. Although NPVs are typically highly species specific, previous work has shown that the specific NPVs of *Spodoptera exigua*, *Spodoptera littoralis* and fall armyworm were able to initiate infection in all three host species (Simon et al., 2004). The most common NPV in Africa is SpexNPV, which is present at high levels in populations of African armyworm *Spodoptera exempta* (Graham et al., 2012; Vilaplana et al., 2010). There is currently no evidence that SpexNPV can infect any other species apart from African armyworm; however, considering its high abundance in Africa (Graham et al., 2012; Vilaplana et al., 2010) and the potential for species spillover from African armyworm to fall armyworm, in this study fall armyworm larvae were screened for the presence of SpexNPV to see if a spillover event had occurred.

*Wolbachia* is a gram‐negative bacterium that is an obligate symbiont of many insect species and can be either parasitic or beneficial (Floate et al., 2006). *Wolbachia* is present within African armyworm populations in Africa, with up to 56% of larvae infected, and it increases larval susceptibility to SpexNPV, leading to higher mortality rates and lower lethal dose thresholds (Graham et al., 2012). *Wolbachia* has not been detected in fall armyworm larvae in the Americas (Dumas et al., 2015). However, spillover events may occur so it is important to establish whether it is present in invasive species as *Wolbachia* can significantly affect population dynamics through mechanisms such as male‐killing and reducing fecundity (Floate et al., 2006; Graham et al., 2012; Graham & Wilson, 2012).

*Metarhizium rileyi* is a widely occurring pathogenic fungus that predominately infects Lepidoptera, with around 60 known susceptible species (Fronza et al., 2017). *M. rileyi* occurs in the United States and South America, however, its prevalence is low and documented cases of it infecting fall armyworm in the field in its native region are rare (Álvarez et al., 2018; Fronza et al., 2017; Ignoffo & Garcia, 1985). *M. rileyi* has been detected in invasive fall armyworm populations in China (Zhou et al., 2020) and India, where it was responsible for around 50% of mortalities in North East India (Firake & Behere, 2020; Mallapur et al., 2018; Sharanabasappa et al., 2019). However, *M. rileyi* has not been officially recorded in Africa on fall armyworm, although fungal infections have been reported in field populations in Malawi and Zambia, and it is present in Africa with frequent infections occurring in African armyworm (Food and Agriculture Organisation, 2018; Rose et al., 2000).

To further improve our current understanding of the population ecology of the host–pathogen interactions of a newly invasive species, this study aimed to establish whether four important entomopathogens are present in fall armyworm in its new territory: SfMNPV, SpexNPV, *M. rileyi* and *Wolbachia*. Following the discovery of SpexNPV in fall armyworm, a bioassay was carried out to determine the impact of SpexNPV infection on fall armyworm. Finally, we determined how the prevalence of SfMNPV (the most common microbial pathogen in invasive fall armyworm in Africa) is affected by a range of environmental factors that have previously been shown to influence pathogen levels, including weather, elevation, time since fall armyworm invasion and the crop development stage.

## 2 MATERIALS AND METHODS

### 2.1 Sample collections

**FIGURE 1:**
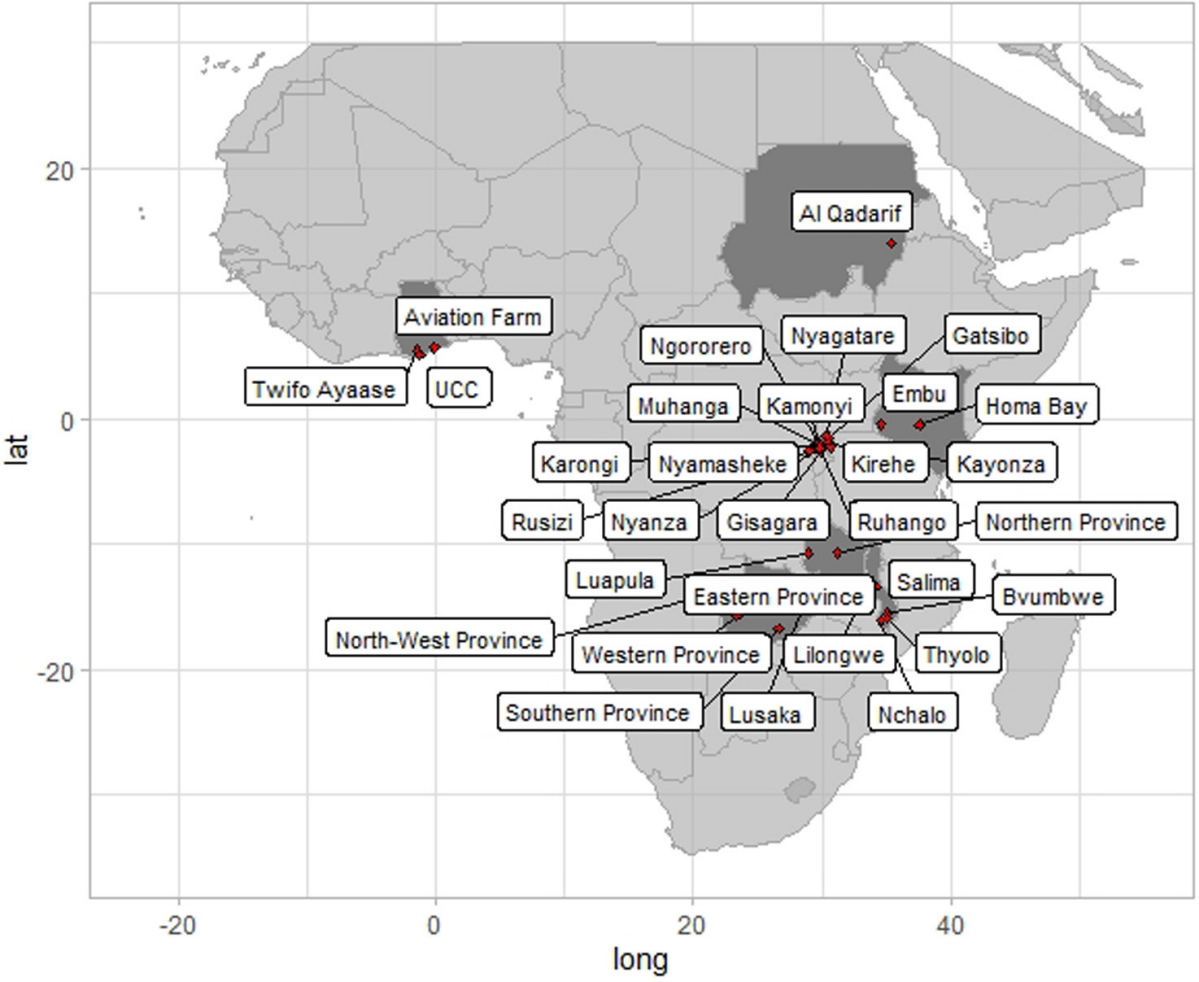
Sampling locations of fall armyworm larvae in Ghana, Kenya, Malawi, Rwanda, Sudan and Zambia.

A total of 496 fall armyworm larvae were collected from maize or sorghum in various locations in Malawi, Rwanda, Kenya, Zambia, Ghana and Sudan between 2017 and 2019 (Table 1, Figure 1). Samples were stored in ethanol at −20°C. Ethical approval and review were not applicable to this study due to working with Lepidopteran invertebrates.

**TABLE 1:** Fall armyworm larvae collection details. All larvae were collected at the third or fourth instar and stored in ethanol at −20°C. Sample size is shown in column *N*. If X (longitude) and Y (latitude) were not recorded, then it was estimated using a central point for the named town or province as this information was needed for geographic, meteorological and temporal analysis of SfMNPV

Country	Location	Collector^a^	*N*	Crop	Date	X	Y
Ghana	Aviation Farm	A	4	Maize	16/10/2017	−0.16	5.68
Ghana	Twifo Ayaase	A	30	Maize	14/08/2017	−1.49	5.47
Ghana	UCC	A	38	Maize	22/12/2017	−1.29	5.11
Kenya	Embu	B	9	Maize	19/06/2019	34.58	−0.43
Kenya	Homa Bay	B	30	Maize	19/06/2019	37.6	−0.49
Malawi	Bvumbwe	C	40	Maize	23/01/2019	35.04	−15.55
Malawi	Lilongwe	C	31	Maize	28/01/2019	33.38	−13.85
Malawi	Nchalo	C	40	Maize	17/09/2018	34.55	−16.16
Malawi	Salima	C	30	Maize	28/01/2019	34.15	−13.4
Malawi	Thyolo	C	40	Maize	17/09/2018	35.07	−15.92
Rwanda	Gatsibo	D	10	Maize	04/05/2017	30.47	−1.58
Rwanda	Gisagara	D	8	Maize	05/05/2017	29.85	−2.59
Rwanda	Kamonyi	D	10	Maize	05/05/2017	29.9	−2.01
Rwanda	Karongi	D	10	Maize	04/05/2017	29.42	−2.16
Rwanda	Kayonza	D	10	Maize	03/05/2017	30.51	−1.91
Rwanda	Kirehe	D	10	Maize	03/05/2017	30.64	−2.26
Rwanda	Muhanga	D	8	Maize	05/05/2017	29.73	−1.96
Rwanda	Ngororero	D	10	Maize	05/05/2017	29.62	−1.86
Rwanda	Nyamasheke	D	10	Maize	03/05/2017	29.17	−2.38
Rwanda	Nyanza	D	9	Maize	05/05/2017	29.75	−2.35
Rwanda	Nyagatare	D	10	Maize	05/05/2017	30.33	−1.29
Rwanda	Ruhango	D	10	Maize	10/05/2017	29.78	−2.31
Rwanda	Rusizi	D	10	Maize	03/05/2017	29.01	−2.58
Sudan	Al Qadarif	F	28	Sorghum	01/09/2017	35.38	14.04
Zambia	Eastern Province	E	4	Maize	18/01/2017	32.42	−12.91
Zambia	Luapula	E	9	Maize	15/05/2017	28.93	−10.71
Zambia	Lusaka	E	15	Maize	20/01/2017	28.32	−15.4
Zambia	Northern Province	E	10	Maize	15/05/2017	31.19	−10.65
Zambia	North‐West Province	E	7	Maize	15/05/2017	25.16	−12.86
Zambia	Southern Province	E	3	Maize	21/01/2017	26.62	−16.73
Zambia	Western Province	E	3	Maize	20/01/2017	23.38	−15.68

a A: Ben Mensah, B: Aislinn Pearson, Sevgan Subramanian and Kentosse Gutu Ouma, C: Donald Kachigamba and Amy Withers, D: Patrick Karangwa and Bellancile Uzayisenga, E: Gilson Chipabika and Miyanda Moonga, F: Guillaume Sneessens.

### 2.2 DNA extraction

DNA was extracted from the posterior half of all larvae (≤25 mg) following the standard protocol for tissue in the Qiagen DNeasy Blood and Tissue kit. DNA was stored in 100 μl buffer AE (10 mM Tris‐Cl 0.5 mM EDTA; pH 9) at −20°C. The protocol was altered slightly for extracting DNA from larvae collected in Sudan, these modifications were 200 μl ATL and an additional 200 μl 1x SSC before incubation and the DNeasy Spin Column was centrifuged at 13,000 RPM.

### 2.3 Microbial detection

Larval fall armyworm DNA samples were screened for the presence of SpexNPV, SfMNPV, *M. rileyi* and *Wolbachia* using standard PCR assays. The primer sequences and cycling parameters for each microbe tested are shown in Table 2. Each PCR reaction was carried out in a 0.2 ml PCR tube and the reagents were 1 μl EasyTaq® Buffer (Transgen biotech), 0.5 μl 10 μM forward primer, 0.5 μl 10 μM reverse primer, 0.2 μl dNTPs (Transgen biotech), 0.05 μl EasyTaq® DNA Polymerase (Transgen biotech), 5.75 μl H_2_O and 1 μl DNA. PCR tests were carried out in duplicate due to low concentrations of pathogen DNA, with the exception of *M. rileyi* which was tested once. PCR products were visualised using agarose gel electrophoresis. If a band was visible in either of the PCR repeats then the sample was recorded as positive for the microbe.

**TABLE 2:** Primer information for each pathogen

Primer	Expected product size (bp)	F primer sequence	R primer sequence	Cycling parameters
*Spodoptera exempta* Nucleopolyhedrovirus: orf 57–58 (Donkersley, unpublished)	300	5′‐GTCGTGCAGTTCCTTGTAGT	5′‐ACAAGACAAACGACAATGTGTG	ID: 95°C 2 min D: 95°C 30 s} A: 60°C 30 s} 30 cyclesE: 68°C 45 s}FE: 68°C 5 min
*Spodoptera frugiperda* Nucleopolyhedrovirus: Sfpg41.1 gene (Simón et al., 2008)	650–750 (some variation between genotypes)	5′‐CGACAATGTCATCGTCTTCG	5′‐ATATGTTAGTGGTGGCGGAC	ID: 95°C 2 min D: 95°C 30 s} A: 52°C 30 s} 30 cyclesE: 68°C 45 s}FE: 68°C 5 min
*Wolbachia* (Zhou et al., 1998)	590–632 (some variation between genotypes)	5′‐TGGTCCAATAAGTGATGAAGAAAC (Wsp81F)	5′‐AAAAATTAAACGCTACTCCA (Wsp691R)	ID: 94°C 5 min D: 94°C 30 s} A: 52°C 30 s} 40 cyclesE: 72°C 45 s}FE: 72°C 5 min
*Metarhizium rileyi* (Tseng et al., 2010)	284	5′‐ CCAAGCCACCAGTCAATTTC (NS1)	5′‐ TATCACCAGCCTCGATCACC (NS2)	ID: 95°C 2 min D: 95°C 30 s} A: 56°C 30 s} 30 cyclesE: 68°C 45 s}FE: 68°C 5 min
Universal fungal primers EF1‐1002 (Stielow et al., 2015)	1000	5′‐ TTCATCAAGAACATGAT	5′‐ GCTATCATCACAATGGACGTTCTTGAAG	ID: 94°C 10 min D: 94°C 1 min} A: 52°C 1 min} 33 cyclesE: 72°C 1 min}FE: 72°C 10 min

### 2.4 Bioassay of SpexNPV in fall armyworm

A laboratory culture of fall armyworm (originally collected from the field in Zambia 3 months previously) was used in bioassays. Fall armyworm neonates were starved for 2 hr in 96‐well plates, and then they were fed a 1 μl droplet of diluted SpexNPV at concentrations of 0 to 10^7^ OBs/ml (occlusion bodies per ml). The viral dose was achieved by diluting with blue food colour, dH_2_O and 30% sugar water to establish whether the virus‐contaminated diet was eaten by the larvae. Fall armyworm were left for 15 min and then neonates with a visibly blue gut were placed into individual diet pots with an excess of artificial diet. Larvae that died within 24 hr of virus challenge were recorded as ‘handling’ deaths and were removed from the analysis. Larvae were monitored daily for mortality and development. Diet pots were changed as necessary.

### 2.5 Identification of overt fungal infections in fall armyworm

Larval samples showing signs of fungal infection were collected on maize in Zambia. Seven larval samples had DNA extracted using the standard protocol for the Qiagen DNeasy Blood and Tissue kit. DNA was amplified using universal fungal primers for EFI, the primer sequences and cycling parameters are shown in Table 2. Amplicons were purified with Microclean and sequenced using the BigDye terminator v3.1 sequencing kit.

### 2.6 Statistical analysis

#### 2.6.1 Identifying geographic differences in the prevalence of *SfMNPV* , *Wolbachia*, *M. rileyi* and SpexNPV

A logistic regression was carried out on the presence/absence data between countries and season of sampling (i.e. wet or dry), and a likelihood ratio test was used to obtain the reported *p* values for each variable. Statistical analyses were performed using R (v. 4.0.3) (R Core Team, 2021).

### 2.7 Geographical, meteorological and temporal data selection

Rainfall and temperature data were obtained from *Copernicus Climate Change Service* and is based on hourly ECMWF ERA5 data at surface level for the development days prior to fall armyworm larvae collection (Boogaard & Grijn, 2021). The average life cycle of fall armyworm from egg to adult varies with temperature (Du Plessis et al., 2020) and so monthly mean temperatures were obtained for the month each sample was collected in. This was then used to estimate development time of fall armyworm (Du Plessis et al., 2020), rounding to the nearest data point available or using the median if the mean temperature was in the middle of two data points. The number of development days was then used to obtain the weather data for the same number of days. For example, at 26°C the mean time of development from egg to larvae was 29 days, so the data included in the model was for the 29 days prior to sample collection. This created the following variables: *Days_with_rain* (The number of days with >1 mm rainfall during the development time of fall armyworm) and *Mean_temp* (The mean temperature of the estimated development time of fall armyworm).

Elevation data were extracted for each sample point using the World_Topo_Map from the ArcGIS Map Service (ArcGIS, 2021) in ArcGIS Pro. This created the following variable: *elevation_m* (the elevation in metres of the sampling location).

The dates of fall armyworm recorded in each country can vary, based on official reports and when the recording was confirmed. Therefore, we ran the model for SfMNPV prevalence for all countries using two measures of time. These were (a) the number of days since fall armyworm had first been recorded in Africa (*days_since_Africa*) and (b) the number of days since fall armyworm had first been recorded in each individual country (*days_since_country*). There was no significant difference between the models (χ^2^_29_ = 89.03, *p* = 0.504) and *days_since_country* had a slightly lower AIC (122.97) than *days_since_Africa* (123.48). Additionally, the number of days since fall armyworm was first recorded in the country showed more variation: Zambia—November 2016, Malawi—December 2016, Kenya and Ghana—March 2017, Rwanda—May 2017 and Sudan—September 2017 (Uzayisenga et al., 2018; Wilson, 2021). Considering this, *days_since_country* was selected as the variable to include in the statistical analysis.

Crop growing season for each country was based on the *FAO Crop Calendar* for Malawi, Ghana, Kenya, Zambia and Sudan, and on the *2017 Seasonal Agricultural Survey* for Rwanda (FAO, 2010; National Institure of Statistics Rwanda, 2017). Rainfall accumulation was calculated in 10‐day blocks from the start date of the crop growing season for each sampling location (maize for Malawi, Rwanda, Ghana, Kenya and Zambia; sorghum for Sudan). For each sampling location, the start of the growing season was designated as the date that >25 mm of rain had accumulated in the previous 10 days. This is because rainfall is directly linked to crop growth, and >25 mm in a 10‐day period typically triggers planting within the growing season for maize (Tadross et al., 2005; Tadross et al., 2009). If this method gave the number of days since the start of the growing season as >5 days longer than the crop cycle period, then the latest date of the crop sowing period was used instead. This gave the following variable: *Days_since_growing_season* (The number of days into the crop growing season it was when sampling occurred).

### 2.8 Modelling the impact on SfMNPV prevalence

Only SfMNPV was modelled due to the relatively low prevalence of the other microbial natural enemies. A PCA was carried out in R (v4.0.3) for the five variables described above (*days_with_rain*, *mean_temp*, *elevation_m*, *days_since_country*, *days_since_growing_season*). Each point on the PCA represents one of 31 sample sites from which larvae were collected between 2017 and 2019 across the six countries (Ghana, Kenya, Malawi, Rwanda, Sudan, Zambia) (Table 1). The three principal components (PCs) that explained the most variation in the data were then extracted and used in a multivariate binomial GLM to determine if they influenced SfMNPV prevalence in Africa. Using the presence and absence values in the model meant that it was weighted based on the number of larvae collected as this varied between sampling locations (Table 1). The three weighted models containing PC1, PC1 + PC2 and PC1, PC2 + PC3 were compared using a Chi^2^ likelihood ratio test in R.

## 3 RESULTS

### 3.1 *M. rileyi* is present in fall armyworm in Zambia, confirmed by sequencing samples with overt fungal infection

**FIGURE 2:**
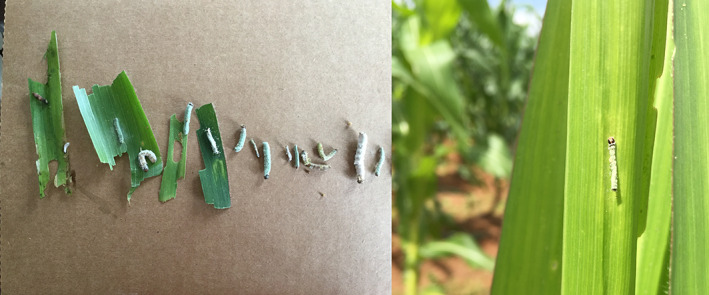
Images of overt fungal infection in fall armyworm in Zambia, that was confirmed by sequencing EF1 to be *Metarhizium rileyi*, (a) All larvae collected with overt signs of fungal infection, (b) Image of larvae on maize crops. Photographs by K. Wilson

Overt fungal infection was observed in 14 fall armyworm larvae while collecting samples in Zambia (Figure 2). Seven of these samples had DNA sequenced and amplified using EF1 primers to determine which fungal species was present. One sample failed to amplify a clear sequence and three samples produced mixed sequences. Of the identifiable sequences, all three were a strong match (99%–100%) to *M. rileyi* confirming that it is present in fall armyworm in Africa (Table 3).

**TABLE 3:** The top three aligned sequences for the successfully amplified EF1 sequences. Images provided by K. Wilson

Sample	Scientific Name	BLAST output for universal fungal sequence (EF1)				
		Length of query sequence	Query Cover	E value	Percent identity	Matching accession number
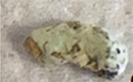	*Metarhizium rileyi*	545	99%	0	100	MH986285.1
	*Metarhizium rileyi*		99%	0	100	KP324764.1
	*Metarhizium rileyi*		99%	0	100	HQ165688.1
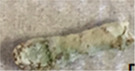	*Metarhizium rileyi*	541	100%	0	100	MH986285.1
	*Metarhizium rileyi*		100%	0	100	KP324764.1
	*Metarhizium rileyi*		100%	0	100	HQ165688.1
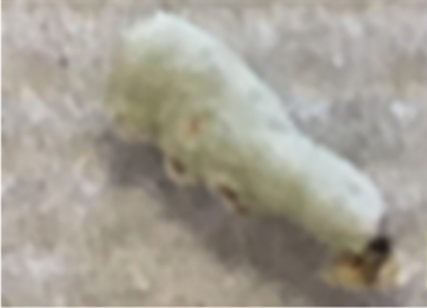	*Metarhizium rileyi*	563	100%	0	100	MH986285.1
	*Metarhizium rileyi*		100%	0	100	KP324764.1
	*Metarhizium rileyi*		100%	0	100	HQ165688.1

### 3.2 The prevalence of *M. rileyi*, *SfMNPV* and SpexNPV varies significantly between countries, however, *Wolbachia* is at equally low prevalence across Africa

**FIGURE 3:**
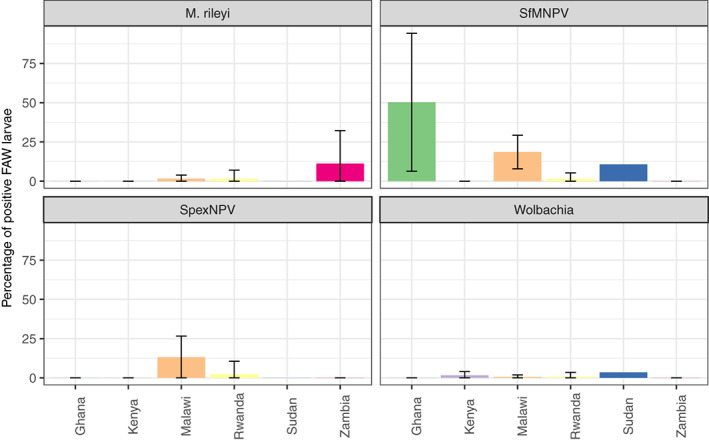
The mean (± standard deviation) percentage of fall armyworm larvae with each microbe in each country.

*M. rileyi* prevalence varied significantly between the countries, ranging from 0% to 12% of samples (*χ* ^2^_5_ = 32.64, *p* = 0.004, Figure 3, Table 4), as did SpexNPV, which ranged from 0% to 13% (*χ* ^2^_5_ = 45.70, *p* < 0.001, Figure 3, Table 4). SfMNPV prevalence significantly varied between the six countries (*χ* ^2^_5_ = 34.38, *p* < 0.001), with the mean percentage of SfMNPV‐positive samples varying from 0% to 31% (Figure 3, Table 4). *Wolbachia*, however, did not significantly vary between the six countries, ranging from 0% to 4% (*χ* ^2^_5_ = 9.27, *p* = 0.472, Figure 3, Table 4).

**TABLE 4:** The prevalence of SfMNPV, SpexNPV, *Metarhizium rileyi* and *Wolbachia* in six African countries. A binomial GLM was carried out to determine if the prevalence of each pathogen significantly varied between countries. Significant *p* values are in italics and bold

Country	*N*	Pathogen presence (%)											
		SfMNPV			SpexNPV			*Wolbachia*			*M. rileyi*		
Ghana	72	30.56			0			0			0		
Kenya	39	0			0			2.56			0		
Malawi	181	17.68			13.26			0.55			1.66		
Rwanda	127	1.57			2.36			0.79			1.57		
Sudan	28	10.71			0			3.57			0		
Zambia	51	0			0			0			11.76		
Difference between countries		*χ* ^2^	*p*	*df*	*χ* ^2^	*p*	*df*	*χ* ^2^	*p*	*df*	*χ* ^2^	*p*	*df*
		34.38	***<0.001***	5	45.70	***<0.001***	5	9.27	0.472	5	32.64	***0.004***	5

### 3.3 The prevalence of *M. rileyi*, *SfMNPV* , SpexNPV and *Wolbachia* does not significantly vary between wet and dry seasons across Africa

In the six countries included in this study the seasons are described as either wet (i.e., heavy, frequent rains) or dry (i.e. infrequent or no rainfall). Prevalence of *M. rileyi*, SfMNPV, SpexNPV and *Wolbachia* did not significantly vary between the wet or dry season (*χ* ^2^_1_ = 29.69, *p* = 0.086, *χ* ^2^_1_ = 31.13, *p* = 0.071, *χ* ^2^_1_ = 45.33, *p* = 0.540 and *χ* ^2^_1_ = 8.10, *p* = 0.279 respectively).

### 3.4 SpexNPV has limited effects on the mortality of fall armyworm

Following the discovery of SpexNPV in fall armyworm in Malawi and Rwanda, a bioassay was performed to determine the effects on fall armyworm. We did not observe any mortality in fall armyworm at any dose tested in this study (0 to 10^7^ occlusion bodies per ml).

### 3.5 SfMNPV distribution is influenced by meteorological, geographical and temporal variables in Africa

There were strong correlations across the five variables assessed in this study (*mean_temp*, *days_with_rain*, *elevation_m*, *day_since_growing_season and days_since_country*), with absolute correlation coefficients, |r|, ranging between 0.162 and 0.905 (Figure S1). This was expected, as crop growing season is inextricably associated with rainfall and temperature, which in turn depends on geographical features such as elevation. Due to the strong correlations between multiple variables, a PCA was used to look at the differences between the countries where samples were collected, and how the distribution of SfMNPV (the most common microbe in African fall armyworm) might be linked to these variables.

The full principal component (PC) details extracted from the PCA, including loadings, contribution and variance are shown in Table 5. PC1 from the PCA explained 59% of the variance and was based predominantly on temperature, rainfall and elevation, suggesting that these factors considerably varied between the larval sampling sites. PC2 from the PCA was influenced strongly by the time since fall armyworm was first recorded in the country and the number of days into the growing season, and this explained a further 18% of the variance between sampling sites. In total the first two PCs explained nearly 80% of the variation in sampling sites across countries.

**TABLE 5:** The results of the principal components analysis on the five environmental variables (*days_with_rain*, *mean_temp*, *elevation_m*, *days_since_country*, *days_since_growing_season*) that could affect SfMNPV prevalence. The principal components (PCs) with an eigenvalue >1 are shaded in grey. The variables with the greatest loadings for each PC (>0.3 or <−0.3) are shown in bold

	PC1		PC2		PC3		PC4		PC5	
	Loading	Contribution	Loading	Contribution	Loading	Contribution	Loading	Contribution	Loading	Contribution
Days_since_growing_season	**−0.36**	13.26	**−0.71**	50.38	**−0.33**	11.08	**0.47**	22.51	0.17	2.77
Days_since_country	−0.29	8.49	**0.51**	26.48	**−0.80**	64.24	−0.04	0.19	0.08	0.69
Elevation_m	**0.52**	26.95	−0.27	7.33	−0.27	7.42	**−0.42**	17.74	**0.64**	40.56
Days_with_rain	**0.50**	25.38	**0.31**	9.71	<0.01	<0.01	**0.77**	59.52	0.23	5.38
Mean_temp	**−0.51**	26.02	0.25	6.09	0.42	17.25	−0.02	0.04	0.71	50.60
Standard deviation	1.72		0.95		0.89		0.51		0.27	
Variance explained	59.29		18.19		15.85		5.22		1.44	
Cumulative variance	59.29		77.48		93.34		98.55		100	

**FIGURE 4:**
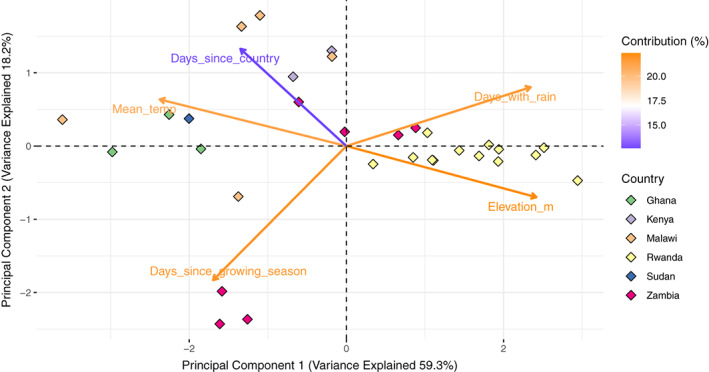
Principal components analysis (PCA) biplot. The PCA was conducted on the five environmental variables (days_with_rain, mean_temp, elevation_m, days_since_country, days_since_growing_season) included in this study. The plot is composed of the first two principal components (PCs) plotted against each other. The colour the variables shows their contribution to the PCs (orange is higher contribution, blue is lower contribution) and the direction shows the correlation with other variables. The six countries are then plotted on to the biplot to show how country variations correspond to the environmental variables studied. Each diamond represents individual sample sites within countries.

Based on the PCs, the three most similar countries were Ghana, Malawi and Kenya, mostly due to similarities in temperature and how long fall armyworm had been in the country prior to larval sample collection (Figure 4, Table 6). Rwanda had a stronger association with higher elevation while samples from Zambia were associated with sample collections later in the growing season (Figure 4, Table 6). This highlights differences between sampling locations and the timing of sample collections (Table 6), and this would add bias to models that include country as a variable when looking at SfMNPV prevalence, therefore PCs were used instead.

**TABLE 6:** The mean and standard deviation (*SD*) for each variable for each country. Sudan only had one sampling location so the mean and standard deviation could not be calculated

Variable	Measurement	Ghana	Kenya	Malawi	Rwanda	Sudan	Zambia
Temperature (°C)	Mean	25.24	21.2	22.74	18.73	27.78	21.98
	*SD*	1.17	1.28	2.89	1.45	NA	1.38
Days with rain	Mean	24.33	36	24.2	46.69	10	28.86
	*SD*	5.77	0	20.36	12.08	NA	15.74
Elevation (m)	Mean	71	1,248.5	768.4	1,633.15	599	1,153.14
	*SD*	66.2	47.38	394.4	220.95	NA	163.41
Days since growing season	Mean	104.67	68.5	80.6	51.23	53	103.14
	*SD*	20.03	17.68	26.24	5.97	NA	60.05
Days since country	Mean	230.33	840	733.8	33.62	1	111.00
	*SD*	65.01	0	71.96	1.85	NA	59.37

PC1 (greatest contributing factors: elevation_m, days_with_rain and mean_temp) and PC2 (greatest contributing factor: days_since_growing_season) were both significantly associated with the prevalence of SfMNPV (*Z* = −4, *p* < 0.001 and *Z* = −2.91, *p* = 0.004 respectively). This suggests that geographical, meteorological and temporal variables all play important roles in determining SfMNPV prevalence in wild FAW populations across Africa. PC3 did not explain significant variation in the prevalence of SfMNPV, suggesting that how long it had been since FAW had first been recorded in the country had a limited effect on SfMNPV prevalence (*Z* = −0.07, *p* = 0.95), and a model comparison showed no significant difference between the model containing PC1 and PC2 and the model containing PC1, PC2 and PC3 (*χ* ^2^_27_ = 72.49, *p* = 0.95).

**FIGURE 5:**
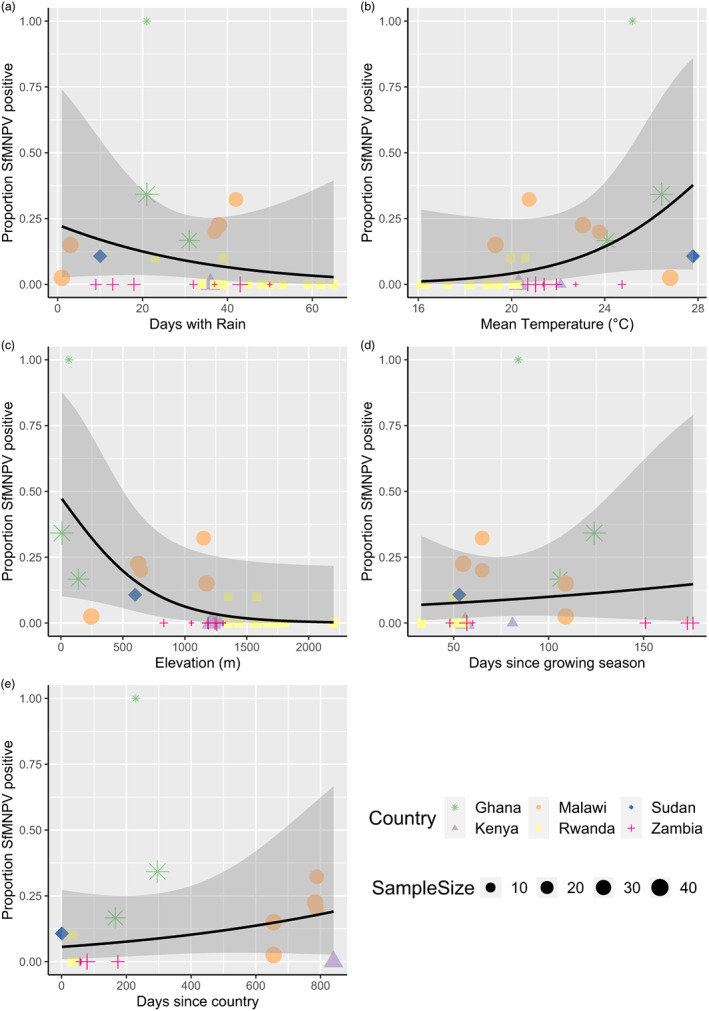
The prevalence of SfMNPV plotted against five different weather variables. (a) Days with rain during larvae development time, (b) Mean temperature during larvae development time, (c) Elevation of sampling location, (d) Days since the growing season began and (e) Days since fall armyworm was first recorded in each country. The shaded area shows the 95% confidence interval. Sample points are proportional to sample size.

Although the variables cannot be modelled alone due to the high correlation, understanding their individual influence on SfMNPV prevalence can help put the results into a broader context that is more applicable. The greatest contributing factors to the highly significant PC1 were rainfall, temperature and elevation (25%, 26% and 27% contribution respectively). Generally, the highest prevalences of SfMNPV were observed in lower elevations which had warmer temperatures and fewer days with rain (Figure 5a–c). However, there is country variation, for example, SfMNPV prevalence increased with more rainy days and declined when the mean temperature was over 25°C in Malawi (Figure 5a,b). The second PC was also significant in explaining SfMNPV and was predominantly based on the temporal variable of days since the growing season began (50% contribution). SfMNPV prevalence was highest between days 50 and 125 of the growing season with a gradual increase during this period, with the lowest prevalences detected at the start and end of the growing season (Figure 5d). It is important to note that while time since first invasion was a key contributor to PC2 (27% contribution), PC3 was predominantly based on this variable (64% contribution) and was not significant in explaining SfMNPV prevalence. There was not a consistent pattern for SfMNPV prevalence and time since first invasion across Africa, however, there was a clear increase in SfMNPV prevalence the longer fall armyworm had been in Ghana and Malawi (Figure 5e).

## 4 DISCUSSION

This study presents evidence that at least four different microbial natural enemies are present in African populations of fall armyworm within 3 years of the species first being documented on the continent, with at least one natural enemy present in each of the six countries we sampled. One of these pathogens, SfMNPV, is well documented in the species' native range, suggesting that some natural enemies were carried over with the starting population of fall armyworm. For two others (SpexNPV and *Wolbachia*) this is the first documented example of fall armyworm encountering these pathogens, and this is likely to have occurred due to spillover from native species, in particular its congener the African armyworm, or due to consuming contaminated plant material due to shared habitats. In this study it was not possible to confirm that the SpexNPV and *Wolbachia* detected were causing active infection in fall armyworm in Africa, however, by revealing that they are present in fall armyworm, this study provides evidence of this and opens the doors for future studies to address this important issue. This is the first confirmed observation of *M. rileyi* infecting fall armyworm in Africa. The prevalence of the most common microbial natural enemy, SfMNPV, varied significantly between the six countries. These findings contribute to the field of invasion ecology by showing that natural enemies can rapidly target newly invasive species, and that not all natural enemies will be left behind when an invasive species moves into a new region.

Invasive populations initially benefit from fewer natural enemies than in their native range meaning they are able to rapidly spread; this is known as the enemy release hypothesis (Cornell & Hawkins, 1993; Torchin & Mitchell, 2004). These findings show that within months of its first invasion, the fall armyworm was exposed to four different microbial natural enemies suggesting that it did not escape them for long. This rapid exposure of the fall armyworm to multiple microbial natural enemies could be due to the closely related species present in Africa (e.g. African armyworm and beet armyworm, *Spodoptera exigua*). The more closely related an invasive species is to native species the more likely they are to be targeted by natural enemies present in the invaded area (Harvey et al., 2012). For example, it was found that the invasive light brown apple moth *Epiphyas postvittana* had a similar abundance of natural enemies in areas it had invaded (California) compared to its native region (Australia), and many of these natural enemies were associated with closely related tortricid species that shared host plants (Bürgi & Mills, 2014). Furthermore, our proposition that two natural enemies (SpexNPV and *Wolbachia*) were present in fall armyworm due to spillover from African armyworm further supports these previous findings, as these spillover events were probably aided by host relatedness. The findings presented here support the growing evidence that the relatedness of invasive species to native ones may influence the extent to which invasive species are able to escape natural enemies due to an increased likelihood of pathogen spillover events.

This study provides the first evidence of SpexNPV spilling over into fall armyworm populations, with SpexNPV found in fall armyworm in Malawi and Rwanda. The interaction between SpexNPV and fall armyworm is not yet fully understood. The laboratory bioassay carried out in this study with SpexNPV in fall armyworm found that no mortality occurred following viral challenge and it was not possible to determine whether the virus was actively replicating in its fall armyworm hosts. NPVs range from monospecific to generalist pathogens, with a wide range of factors affecting their host range, including life‐history traits of their target host (Lepidoptera) and genetic variation in the viruses themselves (Goulson, 2003; Thiem & Cheng, 2009). Before this study, there was no record of SpexNPV infecting any other host species apart from its known target (African armyworm) in the laboratory or field. The prevalence of SpexNPV can be very high in its native host, African armyworm, with 97% of field collected adults testing positive and 21% showing overt signs of infection (Graham et al., 2012), so it is likely that fall armyworm picked up this pathogen naturally following its arrival in Africa.

SpexNPV would not have naturally been in contact with fall armyworm before, therefore this species spillover event happened relatively quickly. This could have been due to co‐infections of both SpexNPV and SfMNPV which can lead to the formation of recombinant NPV with a wider host range, similar to those that occurred naturally between SfMNPV and *Spodoptera litura* NPV in fall armyworm (Barrera et al., 2015; Kondo & Maeda, 1991). Co‐infections of both viruses were detected in larvae from Malawi and Rwanda showing that these do occur in the field. There is some evidence from in vitro experiments that NPVs from different target hosts have higher replication rates in co‐infections, such as with recombinant *Bombyx mori NPV* (BmNPV) and *Autographa californica NPV* (Kondo & Maeda, 1991). However, the opposite has been reported for other NPV co‐infections (Kondo & Maeda, 1991; Shirata et al., 2004). Therefore, this further supports the theory that the enemy release hypothesis may be less applicable to invasive species if there are closely related species that share the same habitat.

Further supporting the increase in natural enemies in invasive species with closely related species present in the new territory, *Wolbachia* was widespread at low prevalence in four of the six countries. *Wolbachia* has not previously been recorded in fall armyworm, and it was not found to be present in a laboratory strain originally collected from Florida (Dumas et al., 2015). However, *Wolbachia* is likely to be able to infect fall armyworm larvae as studies have shown that Wolbachia is capable of infecting and surviving in *S. frugiperda* cells (Sf9 cell line) (Furukawa et al., 2008; Herbert & McGraw, 2018). *Wolbachia* is predominantly transmitted vertically so it is probable that fall armyworm were infected while in Africa due to the absence of *Wolbachia* in fall armyworm in the Americas. Considering they share host plants and co‐infections of SpexNPV and SfMNPV occur, it is likely that fall armyworm and African armyworm have been in close contact since fall armyworm first invaded Africa. *Wolbachia* is present in African armyworm at high prevalence (56%) and was strongly associated with SpexNPV as it increases the susceptibility of African armyworm to the virus (Graham et al., 2012). Therefore, one theory for the detection of *Wolbachia* in fall armyworm proposed here is that a parasitoid vector may have caused the spread of *Wolbachia* from African armyworm to fall armyworm as they share multiple parasitoids, such as *Palexorista zonata* and *Chelonus bifoveolatus* (Rose et al., 2000; Sisay et al., 2018). It has been shown that *Wolbachia* can survive in the mouthparts of a parasitoid vector and be transmitted to other hosts for around 48 hr (Ahmed et al., 2015), and species spillover of *Wolbachia* has previously been recorded between hosts of the same genus, such as Tephritidae fruit flies and Acraea butterflies (Jiggins et al., 2002; Morrow et al., 2015). Alternatively, it is also possible that plant‐mediated transfer of *Wolbachia* could have occurred if infected African armyworm and fall armyworm both fed on the same crop plant (Chrostek et al., 2017).

The most prevalent microbial natural enemy was the baculovirus SfMNPV, and this meant we were able to investigate the factors directly affecting SfMNPV prevalence in more detail. SfMNPV occurs naturally within the Americas, with isolates identified in many countries including Mexico and Colombia at varying prevalences (Cipriano et al., 2013; García‐Banderas et al., 2020; Gómez et al., 2013; Simón et al., 2008). It is therefore likely that some of the first fall armyworm in Africa carried the SfMNPV virus during the incursion event because fall armyworm had not been recorded in Africa before 2016, so it is very unlikely that SfMNPV occurred naturally in Africa prior to this invasion. Further supporting this theory, SfMNPV isolated from fall armyworm in Nigeria was closely related to a naturally occurring SfMNPV isolate collected in Brazil (Wennmann et al., 2021).

In contrast to the enemy release hypothesis, analysis of the principal components suggests that the time since FAW had first been in Africa did not have a significant influence on the prevalence of SfMNPV. This suggests that SfMNPV levels remained relatively constant in the first few years after the fall armyworm reached Africa. Interestingly, there was an increase in SfMNPV with the time since fall armyworm arrived in Ghana and Malawi, however, this effect was not observed in Zambia or Kenya (Figure 5f). Therefore, individuals that spread into Ghana and Malawi may have escaped SfMNPV for a period of time after it first arrived in each country. A similar increase in NPV prevalence over time in spreading populations was observed in another invasive moth, the gypsy moth *Lymantria dispar* in North America (Hajek & Tobin, 2011). This could be due to uninfected individuals having stronger flight capacity than those that are infected which enabled them to disperse further and reach new countries quicker, as has been observed in other migratory Lepidoptera when they were exposed to disease (Bartel et al., 2011; Bradley & Altizer, 2005).

The timing of larval collections relative to planting the crop significantly influenced SfMNPV prevalence with lower levels observed at the start and end of the growing season, and an increase during the middle of the growing season. Population densities of fall armyworm tend to increase as the crop grows but then reduce later into the growing season as crops mature and larvae grow (Caniço et al., 2020). This is because fall armyworm larvae are solitary and cannibalistic, meaning that on mature plants there is usually only one or two larvae present. NPVs persist at higher levels in larger populations, such as SpexNPV in African armyworm, and this is reflected as an evolutionary phenomenon of increased immunity in larvae at higher population densities (Reeson et al., 1998; Vilaplana et al., 2008). Therefore, the lower population densities observed in fall armyworm at the end of the growing season are likely to have reduced transmission of SfMNPV with fewer chances for horizontal transmission. In addition, time into growing season could be a factor in SfMNPV prevalence as plant defences are stronger in mature maize plants, and these defences can influence the effect of infection (Shikano, McCarthy, et al., 2017; Shikano, Shumaker, et al., 2017).

Research has been carried out on the effects of weather variables on SfMNPV used for biocontrol (Fuxa & Richter, 2001), however, this is the first study to consider the effects on naturally occurring viral infections in fall armyworm. Temporal and climatic variables all appear to play an important role in determining variation in SfMNPV prevalence, however, the strong correlation between these variables mean that it is hard to understand their effects individually. Elevation is inextricably linked to weather and was positively correlated with temperature and negatively correlated with rainfall. Considering that elevation, rainfall and temperature made up PC1 that was significantly associated with SfMNPV prevalence, these patterns would suggest that viral prevalence was greatest in lower elevations and warmer temperatures, which are also areas usually associated with less rainfall. However, it is important to consider that behavioural factors relating to these variables could also influence disease prevalence, such as reduced population movements if there is year‐round survival in warmer locations (Cokola et al., 2020). These findings highlight the importance of considering a wide‐range of local factors when investigating the prevalence of natural enemies in invasive species, as these effects of weather and elevation may have led to differences in disease prevalence in fall armyworm populations geographically close together.

Contrary to expectation, this study found that invasive fall armyworm was rapidly exposed to a wide variety of microbial pathogens (SfMNPV, SpexNPV, *M. rileyi* and *Wolbachia*) shortly after arriving in Africa. This shows that not only is fall armyworm in Africa infected with pathogens previously associated with it in its native range, but that it has also been exposed to new microbial pathogens resulting from putative species spillover. This has important implications for our understanding of how disease affects invasive species during the early stages of invasion, and shows that the enemy release hypothesis may not be applicable for long, especially if closely related species are present in the new regions. Furthermore, the work presented here has improved our understanding of the biotic and abiotic factors that influence the natural distribution of microbial natural enemies in Africa. This study has important implications for how we understand the interactions between natural enemies and invasive insects, and reveals the importance of identifying microbial natural enemies in invasive species as soon as possible after they are first detected.

## AUTHORS' CONTRIBUTIONS

A.J.W., C.M.J., J.A.S. and K.W. conceived the ideas and designed the methodology; A.J.W., A.R., J.d.B., P.D., A.J.P., G.C., P.K., B.U., B.A.M., S.A.M., P.O.Y.N., D.K., J.A.S., C.M.J. and K.W. collected the data; A.J.W. led data analyses with support from C.M.J., J.A.S. and K.W.; A.J.W. led the writing of the manuscript. All authors contributed critically to the drafts and gave final approval for publication.

## CONFLICT OF INTEREST

The authors declare no conflict of interest.

## Supporting information


Appendix S1

## Data Availability

Data available from the Dryad Digital Repository https://doi.org/10.5061/dryad.gqnk98sqs (Withers, 2022).
