# Supplementary material for: The distribution of covert microbial natural enemies of a globally invasive crop pest, fall armyworm, in Africa: Enemy release and spillover events
Source: J Anim Ecol. 2022 Jun 22;91(9):1826–41. doi: 10.1111/1365-2656.13760 (PMC9544759; doi:10.1111/1365-2656.13760)
Supplement: Supplementary file 1 — Appendix S1 [file JANE-91-1826-s001.pdf]

Supporting Information for 'The distribution of covert microbial natural enemies of a globally invasive crop pest, fall armyworm, in Africa: enemy-release and spillover events'.

Amy J. Withers[1,2*], Annabel Rice[1], Jolanda de Boer[3], Philip Donkersley[1], Aislinn J. Pearson[2], Gilson Chipabika[4], Patrick Karangwa[5], Bellancile Uzayisenga[5], Benjamin A. Mensah[6], Samuel Adjei Mensah[6], Phillip Obed Yobe Nkunika[7], Donald Kachigamba[8], Judith A. Smith[3], Christopher M. Jones[9,10] and Kenneth Wilson[1*]

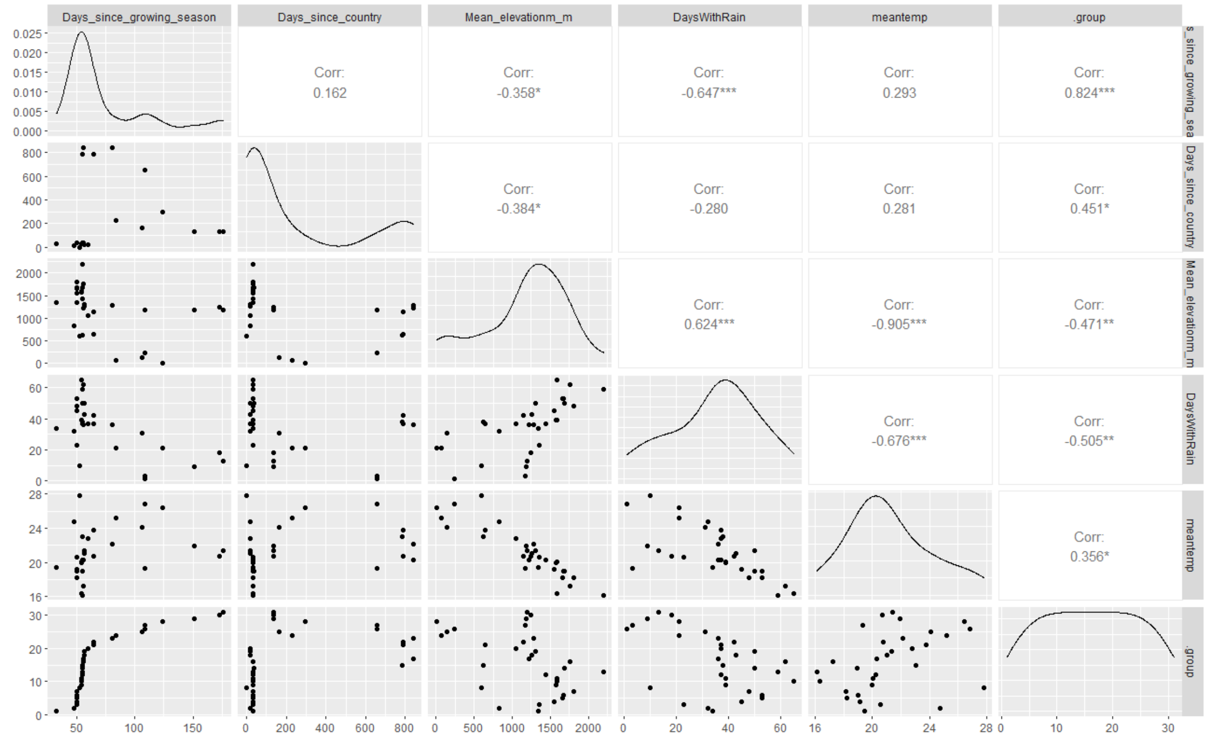

Figure S1: A cross-correlation plot showing the correlations across the five variables in Africa that were assessed in this study (*mean_temp, days_with_rain, elevation_m, day_since_growing_season and days_since_country*).
